# Significant Changes in Plasma Alpha-Synuclein and Beta-Synuclein Levels in Male Children with Autism Spectrum Disorder

**DOI:** 10.1155/2018/4503871

**Published:** 2018-04-08

**Authors:** Wilaiwan Sriwimol, Pornprot Limprasert

**Affiliations:** ^1^Department of Pathology, Faculty of Medicine, Prince of Songkla University, Songkhla 90110, Thailand; ^2^Faculty of Medicine, King Mongkut's Institute of Technology Ladkrabang, Bangkok 10520, Thailand

## Abstract

Alpha-synuclein (*α*-synuclein) and beta-synuclein (*β*-synuclein) are presynaptic proteins playing important roles in neuronal plasticity and synaptic vesicle regulation. To evaluate the association of these two proteins and autism spectrum disorder (ASD), we investigated the plasma *α*-synuclein and *β*-synuclein levels in 39 male children with ASD (2 subgroups: 25 autism and 14 pervasive developmental disorder-not otherwise specified (PDD-NOS)) comparing with 29 sex- and age-matched controls by using enzyme-linked immunosorbent assay (ELISA). We first determined the levels of these two proteins in the ASD subgroups and found that there were no significant differences in both plasma *α*-synuclein and *β*-synuclein levels in the autism and PDD-NOS groups. Thus, we could combine the 2 subgroups into one ASD group. Interestingly, the mean plasma *α*-synuclein level was significantly lower (*P* < 0.001) in the ASD children (10.82 ± 6.46 ng/mL) than in the controls (29.47 ± 18.62 ng/mL), while the mean plasma *β*-synuclein level in the ASD children (1344.19 ± 160.26 ng/mL) was significantly higher (*P* < 0.05) than in the controls (1219.16 ± 177.10 ng/mL). This is the first study examining the associations between *α*-synuclein and *β*-synuclein and male ASD patients. We found that alterations in the plasma *α*-synuclein and *β*-synuclein levels might be implicated in the association between synaptic abnormalities and ASD pathogenesis.

## 1. Introduction

Autism spectrum disorder (ASD) is a group of neurodevelopmental disorders characterized by impaired communication skills, deficits in social interaction, and restricted and stereotyped behaviors [1, 2]. Autistic disorder (autism) and PDD-NOS are two of the four subgroups in ASD [3], which have similar clinical manifestations but differences in severity [1, 2]. ASD is widely accepted to be a complex, heterogeneous, and multifactorial disorder, resulting from both genetic and nongenetic risk factors [2, 4]. Mutations in a number of synaptic proteins and receptor genes in patients with ASD have been reported; therefore alterations of synaptic levels or synapse imbalances have been proposed as a possible contributor to ASD development [5–7]. The genetic contribution in ASD has been extensively studied, whereas proteomic role needs further investigation.

The synuclein protein family is a family of presynaptic proteins localized in the synaptic terminals in the neocortex, hippocampus, striatum, thalamus, and cerebellum regions [8]. *α*-Synuclein and *β*-synuclein are part of this family and they share a characteristic consensus sequence, leading to being the closest in a structural homology [8]. *α*-Synuclein plays a critical role in synaptic functions, including vesicular stabilization, synaptic pool maintenance, synaptic plasticity, and regulations of dopamine synthesis and neurotransmitter release [9–11]. *α*-Synuclein is now thought to be a major component of Lewy bodies, which are a pathological hallmark of neurodegenerative disorders such as Parkinson's disease, multiple system atrophy, and dementia with Lewy bodies [12, 13]. It has been suggested that alterations in the expression of and/or solubility of *α*-synuclein may be a cause of the *α*-synuclein aggregation in these disorders [12–14].

To date, the precise function of *β*-synuclein remains equivocal. It has been shown to be an inhibitor of *α*-synuclein aggregation both* in vivo* [15] and* in vitro* [16]. Beyer and colleagues reported that the expression of *α*-synuclein and *β*-synuclein transcripts in the caudate nucleus was different between Parkinson's disease (PD) and Parkinson's disease with dementia (PDD) [17]. Overexpressed *β*-synuclein mRNA was found in PD while diminished *α*-synuclein mRNA was found in PDD. The specific changes of *α*-synuclein and *β*-synuclein expression and ratio might be involved in a critical role during the development and progression of Lewy bodies (LB) diseases. Another study using mouse model of PD found that the overexpression of *β*-synuclein affected the reduction of *α*-synuclein expression at the protein level [18], indicating a possible correlation in the expression levels between these two synuclein proteins.

The detection of synaptic proteins, including *α*-synuclein and *β*-synuclein, in cerebrospinal fluid (CSF), blood plasma, and serum of both healthy persons and patients has been reported [19, 20]. Two possible mechanisms of *α*-synuclein clearance to maintain the normal levels in the healthy brain have been proposed, via the blood-brain and blood-CSF barriers [10, 21]. In recent years, the measurement of *α*-synuclein in blood plasma has been explored as a potential biomarker for PD. However, the findings are still contradictory, with increased levels, decreased levels, and no significant changes of plasma *α*-synuclein in PD patients compared with normal controls reported from different studies [22–24]. More promising for diagnosis of PD may be phosphorylated *α*-synuclein [25]. Until now, only one study has found low levels of serum *α*-synuclein in ASD patients compared with normal controls [26], while no studies examining *β*-synuclein levels blood samples of ASD patients have been reported.

The present study aimed to examine potential associations between *α*-synuclein and *β*-synuclein levels and male ASD patients by measuring *α*-synuclein and *β*-synuclein protein levels in plasma samples of ASD children and comparing them to those of non-ASD controls by using ELISA.

## 2. Materials and Methods

### 2.1. Subjects

The patient group contained 39 children with ASD, who fulfilled inclusion criteria of the study. They were recruited from child developmental clinics at three university hospitals in Thailand, Songklanagarind, Thammasat, and Ramathibodi hospitals, following diagnosis of ASD based on the Diagnostic and Statistical Manual of Mental Disorders, Fourth Edition (DSM-IV) criteria for autistic disorder (*n* = 25) and PDD-NOS (*n* = 14). Informed consent forms were signed by the parents of each patient. The ASD patients were male with ages ranging from 2 to 8 years, all of whom had normal results from both chromosomal [27] and microarray [28] studies. Their nonverbal IQ scores using the Stanford-Binet Intelligence Scale: Fifth Edition (SB: V) were less than 80 and they also had abnormalities indicated from the Vineland Adaptive Behavior Scale: Interview Edition Survey Form. The control group consisted of 29 sex- and age-matched non-ASD children, who were all recruited from Songklanagarind hospital. Children with other neurodevelopmental disorders (i.e., Down syndrome, intellectual disability, etc.), genetic disorders, history of head injury with loss of consciousness, history of prematurity, and/or low birth weight were excluded from the study. The research protocol was approved by the Ethics Committee, Faculty of Medicine, Prince of Songkla University (REC 59-082-05-2).

### 2.2. Measurement of Plasma *α*-Synuclein and *β*-Synuclein Levels

Nonhemolyzed, nonhyperlipidemic, and nonicteric lithium-heparinized plasma was used in this study. Multiple aliquots of all plasma samples were stored at −80°C until required. The plasma concentrations of *α*-synuclein and *β*-synuclein were determined by double-antibody sandwich ELISA, using commercial kits according to the manufacturers' instructions (Human *α*-Synuclein Assay Kit-IBL, Code Number 27740, and Human Beta-Synuclein ELISA Kit-FIVE photon Biochemicals, Part Number hSNCB-Biotin (96T)). The plasma samples were prepared to tenfold dilution with sample buffer supplied with the kits in order to obtain adequate concentrations to measure. To increase accuracy, all plasma samples were analyzed in two independent experiments, with each experiment also performed in duplicate.

### 2.3. Statistical Analysis

Categorical variables were analyzed as percentages and continuous variables were analyzed using mean ± standard deviation and range with 95% confidence intervals. The quantitative data were compared using Student's *t*-test for independent variables. All statistical analyses were performed using the SPSS program, version 21.0, and statistical significance was defined as *P* < 0.05.

## 3. Results

### 3.1. Demographic and Clinical Details of ASD Patients and Age-Matched Controls

The demographic and clinical characteristics of the ASD patients and controls are shown in Table 1. The control group contained 29 males with a mean age of 5.04 ± 2.06 years while the ASD patient group consisted of 39 males with a mean age of 4.20 ± 1.37 years, the difference not being statistically significant. The mean nonverbal IQ of the ASD patients was 58.85 ± 10.44, and they were classified into severe deficit (*n* = 1, 2.56%), moderate deficit (*n* = 21, 53.85%), mild deficit (*n* = 16, 41.03%), and adequate (*n* = 1, 2.56%) groups based on Vineland test scores. The ASD patients were subdivided into two subgroups, the autism (*n* = 25, 64.10%) and PDD-NOS (*n* = 14, 35.90%) groups. The mean ages were not statistically significantly different between these two groups (3.90 ± 1.20 years versus 4.73 ± 1.53 years for autism and PDD-NOS, resp.). However, the mean nonverbal IQs were statistically significantly different between the groups (56.32 ± 10.09 versus 63.36 ± 9.83, resp.; *P* < 0.05).

### 3.2. Plasma Levels of *α*-Synuclein and *β*-Synuclein in the ASD Subgroups: Autism and PDD-NOS

**Figure 1:**
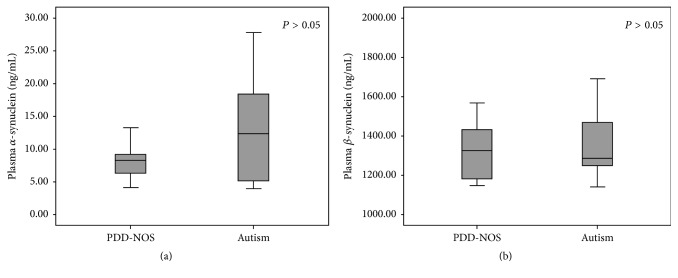
Boxplots comparing the plasma levels of *α*-synuclein (a) and *β*-synuclein (b) between the PDD-NOD and autism groups. Data boxes are shown as median and 25th/75th percentile.

The mean plasma *α*-synuclein and *β*-synuclein levels in the autism group were 12.04 ± 7.35 ng/mL (95% CI 9.01–15.07) and 1358.22 ± 173.14 ng/mL (95% CI 1286.75–1429.69), respectively, and 8.65 ± 3.80 ng/mL (95% CI 6.45–10.85) and 1319.14 ± 136.67 ng/mL (95% CI 1240.33–1398.05), respectively, in the PDD-NOS group. Subgroup analysis found no statistically significant differences in the mean plasma *α*-synuclein and *β*-synuclein levels between these subgroups (Table 1 and Figures 1(a) and 1(b)).

### 3.3. Plasma Levels of *α*-Synuclein and *β*-Synuclein in the Vineland Subgroups of ASD Patients

The moderate (*n* = 21) and mild (*n* = 16) deficits are two major subgroups of the 39 ASD patients (Table 1) when classifying based on Vineland test scores. The mean plasma *α*-synuclein and *β*-synuclein levels in the moderate deficit group were 11.51 ± 5.58 ng/mL (95% CI 8.97–14.05) and 1321.10 ± 129.11 ng/mL (95% CI 1262.33–1379.86), respectively, and 10.67 ± 7.67 ng/mL (95% CI 6.58–14.75) and 1362.28 ± 194.75 ng/mL (95% CI 1258.51–1466.05), respectively, in the mild deficit group. Vineland subgroups analysis had no statistically significant differences in the mean plasma *α*-synuclein and *β*-synuclein levels between these moderate and mild deficits subgroups.

### 3.4. Plasma Levels of *α*-Synuclein and *β*-Synuclein in Male ASD Patients and Controls

**Figure 2:**
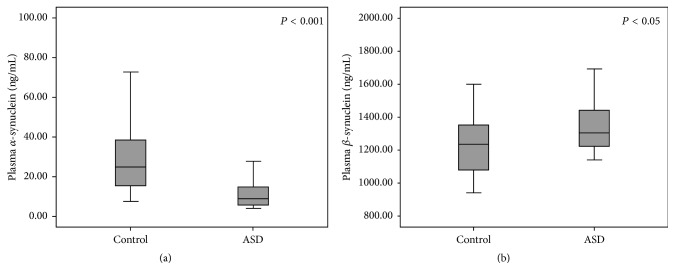
Boxplots comparing the plasma levels of *α*-synuclein (a) and *β*-synuclein (b) between the controls and ASD patients. Data boxes are shown as median and 25th/75th percentile.

The mean plasma *α*-synuclein levels of the male ASD patients and the male controls were 10.82 ± 6.46 ng/mL (95% CI 8.73–12.92) and 29.47 ± 18.62 ng/mL (95% CI 22.39–36.56), respectively (Table 1 and Figure 2(a)). The mean plasma *α*-synuclein level was significantly lower in the children with ASD as compared to the controls (*P* < 0.001). Additionally, the mean plasma *β*-synuclein levels in the ASD patients and the controls were 1344.19 ± 160.26 ng/mL (95% CI 1292.24–1396.14) and 1219.16 ± 177.10 ng/mL (95% CI 1151.79–1286.52), respectively (Table 1 and Figure 2(b)), significantly higher in the ASD group (*P* < 0.05). Pearson correlation analysis showed no statistically significant correlation between plasma *α*-synuclein and *β*-synuclein levels of both ASD (*r* = 0.059, *n* = 39, *P* > 0.05) and control (*r* = 0.203, *n* = 29, *P* > 0.05) groups.

## 4. Discussion

The present study is the first report to elucidate the association of both *α*-synuclein and *β*-synuclein proteins with ASD patients compared to sex- and age-matched non-ASD controls. Although the *α*-synuclein and *β*-synuclein proteins are predominantly expressed in presynaptic terminals at several regions of the brain, earlier studies have found that these proteins could be detected in human bodily fluids including CSF and blood plasma [19, 20]. It is believed that *α*-synuclein is regulated and released outside the central nervous system (CNS) via the blood-brain and blood-CSF barriers for maintaining normal levels in the healthy brain [21, 29]. Recently, a number of studies have attempted to determine the feasibility of using plasma *α*-synuclein level as a biological marker for diagnosis of neurodegenerative disorders, especially Parkinson's disease [22–24].

In this work, we found significant differences in both plasma *α*-synuclein and *β*-synuclein levels between the male ASD and control groups. The plasma *α*-synuclein level was significantly lower whereas the plasma *β*-synuclein level was significantly higher in children with ASD than in the controls. Our results agree with the study of Kadak et al. [26], which also showed the decreased concentrations of serum *α*-synuclein in the ASD patients. Other previous reports have found that *α*-synuclein plays an important role in synaptic functions, contributing to normal neuronal network activity and connectivity of the healthy brain [9–11]. Additionally, another study has suggested that dopamine homeostasis and neurotransmitter release may be controlled by *α*-synuclein level [11]. Moreover, it was recently reported that the abnormal levels of *α*-synuclein could affect CADPS2 mRNA expression, causing alterations to synaptic functions [30]. Therefore, we hypothesize that neural overconnectivity and synapse alteration reported in the pathogenesis of ASD might result from abnormal levels of *α*-synuclein.

The *α*-synuclein protein was expressed from the human *α*-synuclein gene* (SNCA)*, and a number of studies have shown that the *α*-synuclein expression in PD could be affected by point mutations, duplications, or triplications of the* SNCA* gene [31–33]. Thus the reduction of protein expression from some abnormalities of the* SNCA* gene might be the cause of decreased plasma *α*-synuclein levels. Additionally, gene triplication and overexpression have been found to induce the aggregation of *α*-synuclein protein [34, 35], and the aggregation may decrease monomeric *α*-synuclein level in some neurodegeneration disorders. Recently, there has been evidence of development regression and loss of previously acquired skills and abilities in a significant number of children with ASD, suggestive of neurodegeneration in ASD [36]; however, whether *α*-synuclein aggregation could occur in ASD pathogenesis resulting in the reduction of plasma level is still an open question.

Various works have shown that the clearance of *α*-synuclein outside the CNS is an important mechanism in maintaining normal levels in the healthy brain [21, 29]. Various studies have shown that ASD patients have abnormal brain growth [37, 38], which may affect the blood-brain and/or blood-CSF barriers. Therefore, abnormalities of *α*-synuclein clearance to the CSF and blood may be another possible cause of decreased levels of *α*-synuclein in children with ASD. Although we hypothesize that ASD may be associated with lower plasma *α*-synuclein from impairment of brain function and growth, alternative explanations should be considered and further studied since altered *α*-synuclein levels may be affected by being released from other tissues [23].

In our study, the children with ASD showed significant increased plasma *β*-synuclein levels compared to the non-ASD controls. We cannot clearly explain why the plasma *β*-synuclein levels in ASD were higher, but it is possible that some factor in ASD children could induce the overexpression of *β*-synuclein, and further elucidation of the exact cause or mechanism of these elevated plasma *β*-synuclein levels will make further interesting studies. A PD study using a mouse model found that overexpression of *β*-synuclein was associated with reduced expression of *α*-synuclein [18]. It is another possibility that increased plasma *β*-synuclein level may be a compensatory effect for *α*-synuclein reduction.

Up to date, the diagnosis of ASD is based on behavioral signs and symptoms that are difficult to assess in very young children. The clinical heterogeneity in ASD children has encouraged increasing interest in biomarkers as potentially useful indicators for diagnosis and treatment. Recently, a number of studies have attempted to identify potential blood-based biomarkers based on neurophysiological and biochemical alterations, for example, brain-derived neurotrophic factor (BDNF) [39–41], neurotrophin-4 (NT-4) [41], serotonin (5-HT) [42, 43], neopterin [44], and the glial fibrillary acidic protein [45]. However, no biomarkers to date have shown high sensitivity and specificity. The present study found an association of plasma *α*-synuclein and *β*-synuclein alterations with ASD; thus these proteins should be considered as potential biomarkers.

## 5. Conclusion

In conclusion, abnormal plasma levels of *α*-synuclein and *β*-synuclein proteins in male ASD children might indicate the involvement of synaptic dysfunctions in the pathogenesis of ASD. To clarify our findings, further elucidation regarding the *α*-synuclein and *β*-synuclein systems should be considered, especially studies examining the levels of* SNCA* and* SNCB *mRNA at various brain regions using an ASD mouse model. In addition, further studies in larger and different case-control studies are needed to provide further data on these issues.

## Figures and Tables

**Table 1:** Demographic and clinical features and plasma *α*-synuclein and *β*-synuclein levels of the ASD patients and controls.

	Controls (*n* = 29)	ASD patients		
		All ASD patients (*n* = 39)	PDD-NOS (*n* = 14)	Autism (*n* = 25)
Age (mean ± SD)	5.04 ± 2.06	4.20 ± 1.37	4.73 ± 1.53	3.90 ± 1.20
Nonverbal IQ (mean ± SD)	ND	58.85 ± 10.44	63.36 ± 9.83	56.32 ± 10.09^c^
Vineland test [*n* (%)]	ND			
Severe deficit		1 (2.56)	0 (0)	1 (4)
Moderate deficit		21 (53.85)	9 (64.29)	12 (48)
Mild deficit		16 (41.03)	4 (28.57)	12 (48)
Adequate		1 (2.56)	1 (7.14)	0 (0)
Plasma *α*-synuclein (ng/mL)				
Mean ± SD	29.47 ± 18.62	10.82 ± 6.46^a^	8.65 ± 3.80	12.04 ± 7.35
Range (95% CI)	22.39–36.56	8.73–12.92	6.45–10.85	9.01–15.07
Plasma *β*-synuclein (ng/mL)				
Mean ± SD	1219.16 ± 177.10	1344.19 ± 160.26^b^	1319.14 ± 136.67	1358.22 ± 173.14
Range (95% CI)	1151.79–1286.52	1292.24–1396.14	1240.33–1398.05	1286.75–1429.69

ASD: autism spectrum disorder, PDD-NOS: pervasive developmental disorder-not otherwise specified, IQ: intelligence quotient, CI: confidence interval, and ND: not done; ^a^*P* < 0.001 compared to controls, ^b^*P* < 0.05 compared to controls, and ^c^*P* < 0.05 compared to PDD-NOS group.

## Data Availability

The data used to support the findings of this study are available from the corresponding author upon request.
